# Convolutional neural networks for improving image quality with noisy PET data

**DOI:** 10.1186/s13550-020-00695-1

**Published:** 2020-09-21

**Authors:** Josh Schaefferkoetter, Jianhua Yan, Claudia Ortega, Andrew Sertic, Eli Lechtman, Yael Eshet, Ur Metser, Patrick Veit-Haibach

**Affiliations:** 1 Joint Department of Medical Imaging, Princess Margaret Hospital, University Health Network, Mount Sinai Hospital and Women’s College Hospital, University of Toronto, 610 University Ave, Toronto, ON M5G 2 M9 Canada; 2 Siemens Medical Solutions USA, Inc., 810 Innovation Drive, Knoxville, TN 37932 USA; 3 Shanghai Key Laboratory for Molecular Imaging, Shanghai University of Medicine and Health Sciences, Shanghai, 201318 China

**Keywords:** Deep learning, PET image quality, Lesion detection

## Abstract

**Goal:**

PET is a relatively noisy process compared to other imaging modalities, and sparsity of acquisition data leads to noise in the images. Recent work has focused on machine learning techniques to improve PET images, and this study investigates a deep learning approach to improve the quality of reconstructed image volumes through denoising by a 3D convolution neural network. Potential improvements were evaluated within a clinical context by physician performance in a reading task.

**Methods:**

A wide range of controlled noise levels was emulated from a set of chest PET data in patients with lung cancer, and a convolutional neural network was trained to denoise the reconstructed images using the full-count reconstructions as the ground truth. The benefits, over conventional Gaussian smoothing, were quantified across all noise levels by observer performance in an image ranking and lesion detection task.

**Results:**

The CNN-denoised images were generally ranked by the physicians equal to or better than the Gaussian-smoothed images for all count levels, with the largest effects observed in the lowest-count image sets. For the CNN-denoised images, overall lesion contrast recovery was 60% and 90% at the 1 and 20 million count levels, respectively. Notwithstanding the reduced lesion contrast recovery in noisy data, the CNN-denoised images also yielded better lesion detectability in low count levels. For example, at 1 million true counts, the average true positive detection rate was around 40% for the CNN-denoised images and 30% for the smoothed images.

**Conclusion:**

Significant improvements were found for CNN-denoising for very noisy images, and to some degree for all noise levels. The technique presented here offered however limited benefit for detection performance for images at the count levels routinely encountered in the clinic.

## Key points

Question: Can a CNN be reliably used to denoise PET images and improve image quality for clinical use?

Pertinent findings: This technique offers clear qualitative and quantitative benefits at high noise levels. Some qualitative benefits were also observed in lower noise too, but it yielded limited benefits for detectability performance at clinically routine count levels.

Implications for patient care: This approach, and other deep learning-based methods, might be well suited to improve current or potentially future protocols yielding noisy data, e.g., low dose PET for lung screening.

## Introduction

Positron emission tomography (PET) is an inherently noisy imaging modality. Each sinogram projection bin of a routine PET acquisition contains only a few coincident events. This situation becomes even more problematic in very low-count conditions, e.g., low radiotracer dose [1, 2], short scan time [3], or quick dynamic framing [4, 5]. The reconstruction task is therefore ill-posed, and current algorithms seek to recover the true underlying activity distribution by generating an image representing the most likely estimate given the measured data. Notwithstanding the significant improvements to image quality realized by reconstruction techniques like time-of-flight [6] and resolution modeling [7], reconstructions of sparse PET data can still produce images of poor quality with possibly limited clinical use.

Due, in part, to advances in processing hardware, the past decade has seen a surge in research focused on machine learning and artificial intelligence. Deep learning and convolutional neural networks (CNNs), in particular, have produced state-of-the-art results in the fields of object detection [8, 9], classification [10, 11], image segmentation [12, 13], speech recognition [14], and image generation with adversarial networks [15, 16]. Recent years have also seen the emergence of AI for various applications in medical imaging, including organ segmentation [17, 18], image denoising [19–21], and cancer detection [22].

This work presents an experiment designed to evaluate the use of CNNs for improving the noise properties of PET images reconstructed from low-count data in lung cancer patients. The noise in PET images is generally assumed to follow Gaussian and/or Poisson distributions, and deep learning is especially well positioned to address this since the characteristic features of the noise, regardless of the assumed model, are inherently learned through training. Several techniques have previously been applied successfully for denoising PET images [23–26], to date however, there are very few studies exploring CNNs which handle 3D data. Due to the volumetric nature of data, it is expected that the performance of CNNs could be improved [27].

The objectives of the current study are: (1) to assess the performance of a dedicated 3D CNN trained to improve clinical PET images in a wide range of noise conditions, (2) to investigate the benefits and limitations of this approach in general image quality as well as in lesion detection and characterization, and (3) to evaluate the clinical reading performance in CNN-denoised images vs. standard clinically used images.

## Methods

### Convolutional neural network

**Fig. 1:**
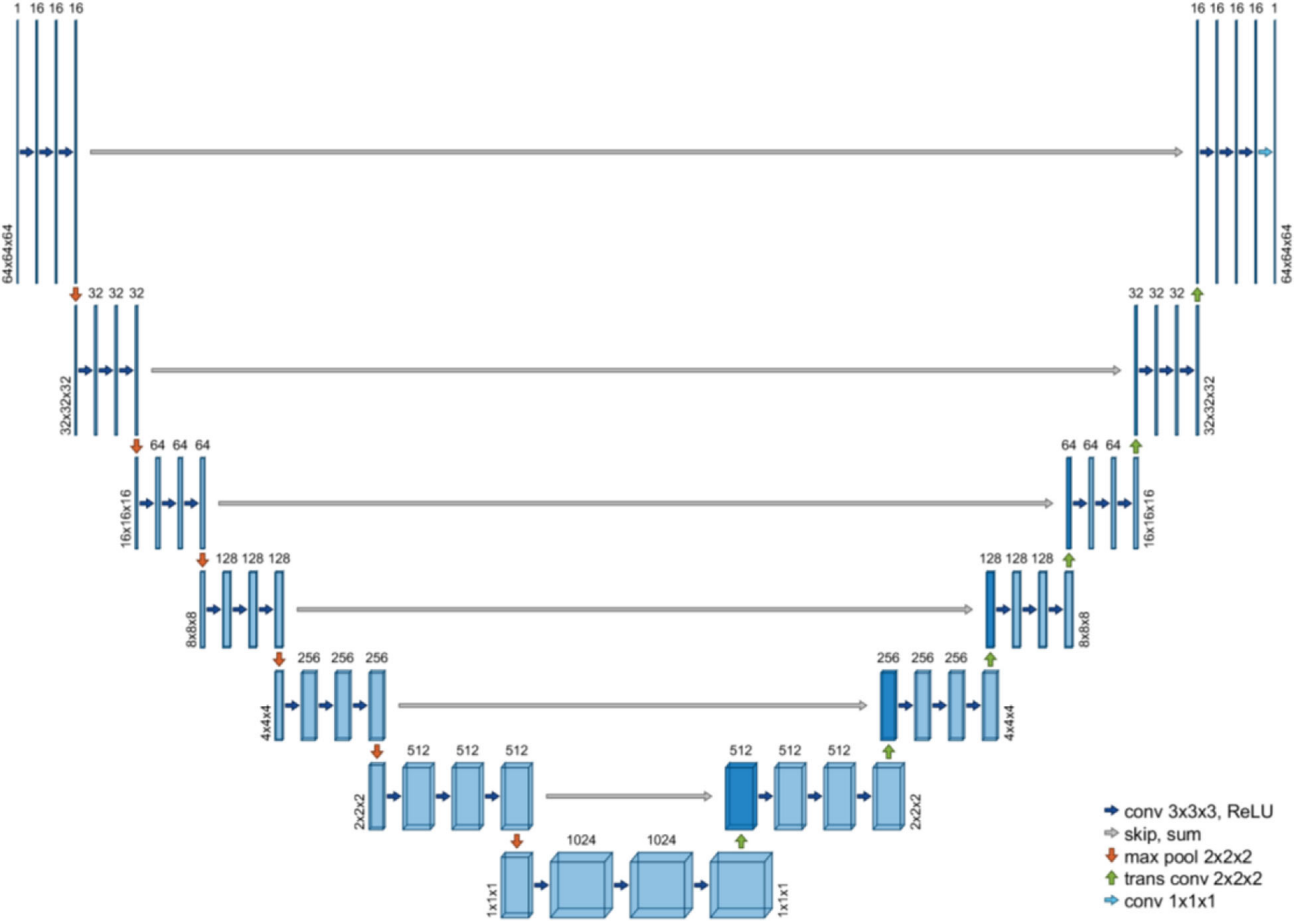
The CNN used in this study followed a U-Net architecture with encoding and decoding paths, connected with direct skip-sum layers at each resolution block

A convolutional neural network framework, compatible for 3D data, was developed in C++ and built on the CUDA deep learning libraries. The network architecture, seen in Fig. 1, was similar to U-Net [17], with symmetric contracting (encoding) and expanding (decoding) paths. The contracting path comprised 6 blocks of 3 convolution layers followed by a max pooling layer, and the expanding path comprised 6 blocks of 3 convolution layers followed by a strided transposed convolution layer. There was additionally a vertex block with 3 convolution layers followed by a strided transposed convolution layer. To improve training stability and preserve fine details at higher resolutions, the output of the last convolution layer in each block on the contracting path was added directly to the input of the corresponding resolution block on the expanding path. Instance normalization [28] was performed before every nonlinearity. Rectified linear activation functions were used in all layers except for the output layer, which used no activation (or normalization)—hence, the range of network output values was not bounded.

### Patient PET data

The CNN was trained with real patient data. The training data were PET images acquired in a cohort of 31 non-small cell lung cancer patients—nine were selected for training. All patient data were obtained on a Biograph mCT (Siemens Healthcare Molecular Imaging) after an uptake period of 60 min and after injection of 225.3 ± 5.6 (214.6–233.1) MBq of [^18^F]-FDG. All data were acquired in list mode. The patients were scanned with 1 or 2 bed positions over the torso, for 10 min per bed, resulting in 132.7 ± 57.6 million true counts (prompts minus randoms) per bed. All patients provided written consent as required by the NHG Domain Specific Review Board.

The full PET datasets were used to emulate lower count levels through random list mode decimation [29] according to 9 predefined levels: 20, 15, 10, 7.5, 5, 2, 1, 0.5, and 0.25 million trues—independent realizations were generated at each count level, resulting in approximately 270 reduced-count datasets for every full-count set. All images were then reconstructed with OSEM, corrected for attenuation and scatter and incorporating time-of-flight information and point-response modeling, for 2 iterations and 21 subsets. For each bed, the final image matrix was 400 × 400 × 109, with voxel dimensions 2.0863 × 2.0863 × 2.0313 mm.

### Network training

The emulated noisy images, along with their corresponding “ground truth” (full-count) images, made up the paired supervised training sets. For each network training epoch, 1600 samples were generated by randomly dividing the training image data into 64 × 64 × 64 volume patches—the noisy slices at the axial extremes of each bed position were not used for training. Each 3D patch sample was normalized to zero mean and unit variance prior to training. These normalization factors were saved for 2 purposes: to scale the corresponding low-noise output label by the same amounts and to invert the transformation in the output after network inference. Under this approach, the network only “saw” data which was already “instance normalized.” This way, a very noisy input would be mapped to an output with smaller variance than that of its less noisy correlate, but after the final scaling, both would be quantitatively accurate. Training was performed using a sample batch size of 16; the Adam optimizer was used to minimize the L2-norm (mean squared error) loss. The learning rate was initialized at 0.01 and decayed by 5% after each epoch, and L2-norm weight regularization was used. Prior to training, the data from two patients were removed from the training population for validation. The CNN was trained with the images from all count levels and was tested continually on the validation set to monitor the training performance—training stopped when the loss curve within the validation set was observed to flatten.

### Clinical evaluation

PET image quality and lesion detection tasks were developed and presented to 3 physicians with image reading experience (3, 3, and 9 years). The acquisition data from twenty patients (not used for training) were selected for the viewing tasks and reconstructed once with the full data, i.e., the ground truth image, and twice at each of the reduced count levels 20, 10, 5, 2, and 1 million trues, which were equivalent to 90, 45, 23, 9, and 5 s scan times. For every reduced count reconstruction, one image was denoised by the trained CNN and the other underwent conventional Gaussian smoothing by a 3-mm FWHM filter—this approach yielded 11 images per patient. The full-count images were also smoothed by a 3-mm filter. Throughout this manuscript, the images post-processed only by Gaussian smoothing are referred to as “original.” For the purposes of this study, only 1 smoothing filter was investigated—this was regarded by our physicians as a good compromised between noise reduction and resolution.

Prior to the observer evaluations, the capacity of our trained CNN to recover lesion contrast was investigated and quantified. Sixty-five lesions were identified and delineated on the full-count PET images by 40% max-threshold contouring, resulting in 65 VOIs ranging in volume from 0.2 to 61.5 cm^3^ (mean 4.6 ± 9.3, median 1.3 cm^3^). The voxel statistics within these VOIs were compared between the CNN-denoised and the smoothed original image sets for every count level.

For the qualitative evaluation, each observer read the total pool of 220 images, rating each on a 4-point scale according to 3 criteria: image noise, image sharpness, and general image quality—this scheme was similar to previously published criteria [30]. Additionally, for each patient full-count image, the observer selected locations of liver, lung, and blood pool (left ventricle or aorta) to be used subsequently for volume-of-interest (VOI) analyses, in which spherical VOIs, of radius 10 mm for liver and lung and 5 mm for blood pool, were defined and propagated throughout the reduced-count images for each patient.

For the lesion detection evaluation, 12 (of the 20) patients were included in the task. These patients were included in the detection task because they were identified on the full-count images as having isolated and clear pathological foci within the mediastinum and/or lung parenchyma.

The physicians were instructed to locate and rank, also on a 4-point scale, the detectability of every suspicious lesion in each of the 132 image volumes—the display was continuously updated to highlight all previously selected lesions within the current subject so as to avoid repeated selections. Performance in each reduced-count image was evaluated relative to the performance in the corresponding full-count image and reported in terms of true positive rate (TPR), false positive rate (FPR), false negative rate (FNR), positive predictive value (PPV), and sensitivity. The area under the receiver operating characteristic curve (ROC-AUC) was also calculated from the detection ratings.

## Results

### Lesion contrast

**Fig. 2:**
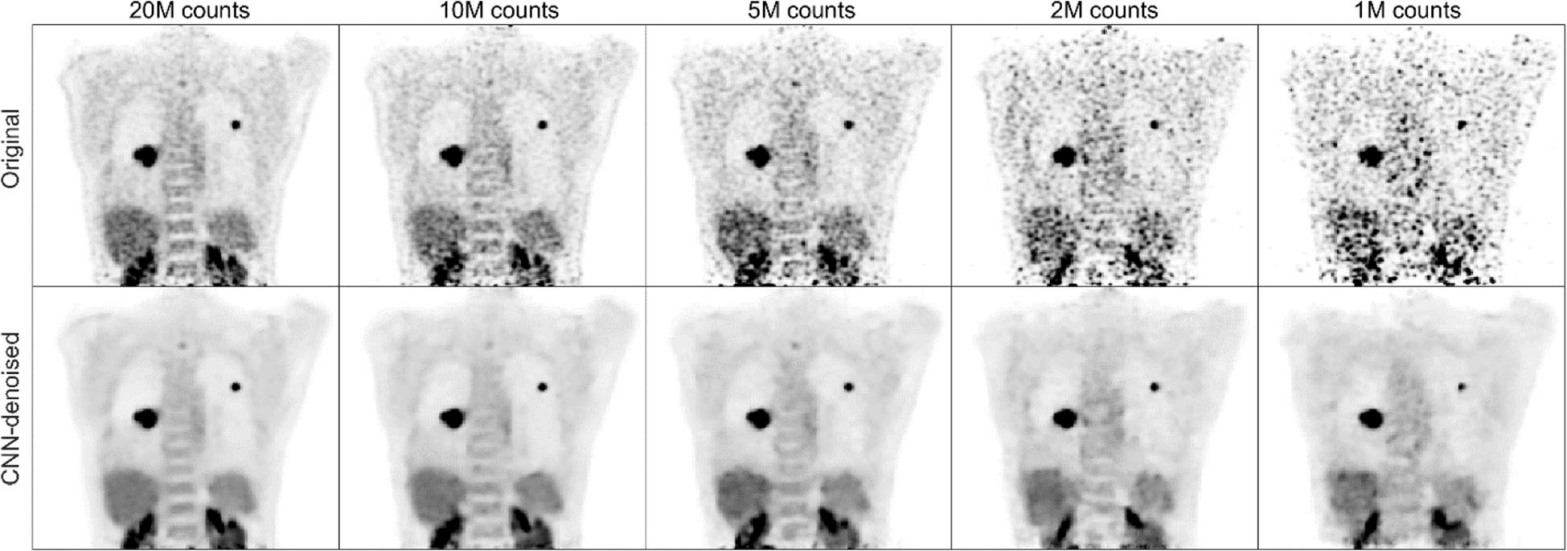
After training completed, the CNN produced outputs with improved noise properties, relative to the original image volumes—inter-voxel spatial variance was reduced and anatomical boundaries were generally preserved

The presented methods were evaluated within all emulated noise levels. After the network training converged within the validation set, the CNN demonstrated the capacity to learn to reproduce the latent distributions from which the noisy data originated. This image denoising manifested not only as a smoothing effect, but as local image regularization, i.e., relatively uniform regions with clear anatomical boundaries. The quality of the CNN-denoised images appeared equal or superior to their corresponding original images at every count level; as seen in Fig. 2, general anatomical structures were more clearly defined.

**Fig. 3:**
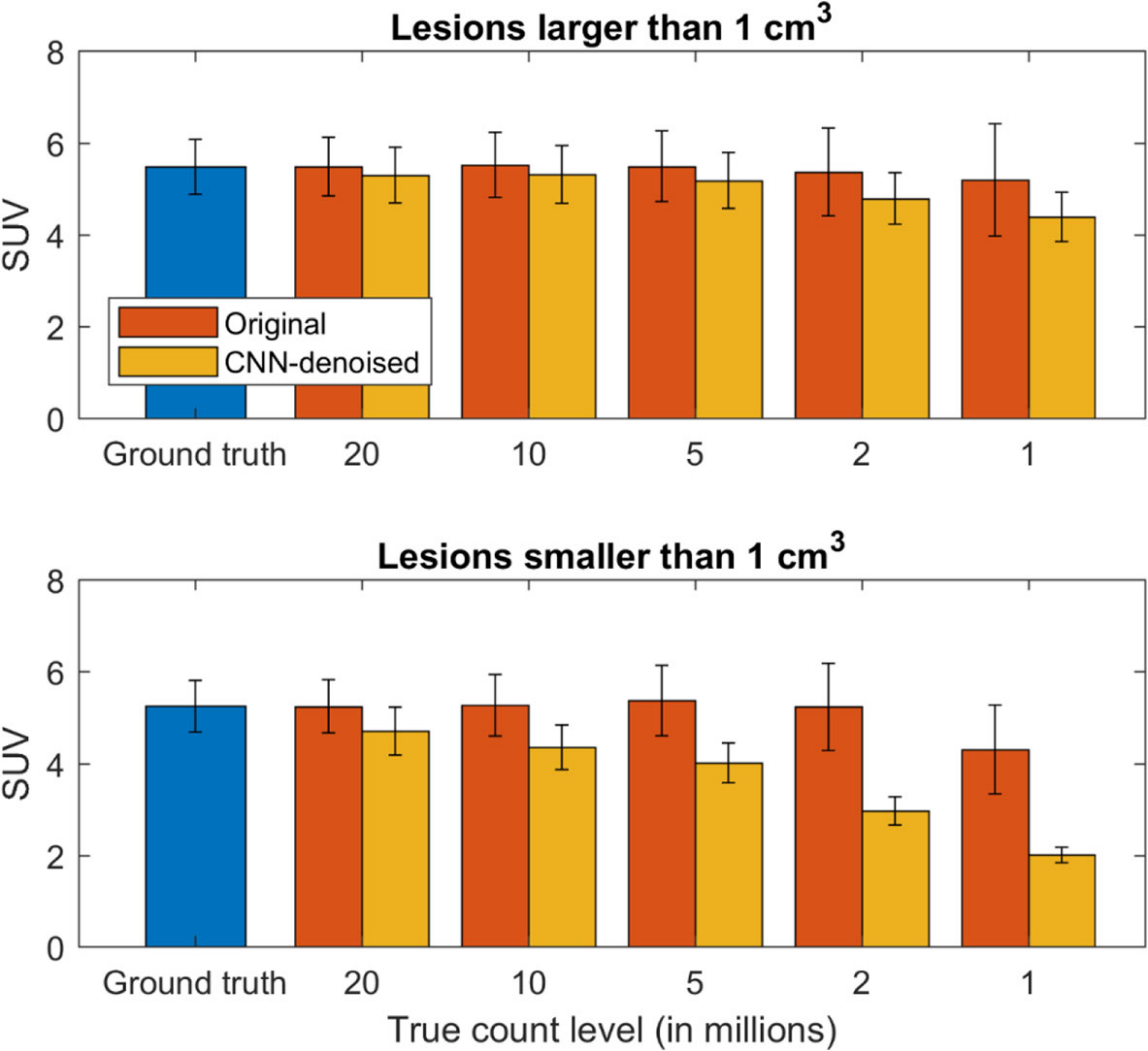
Mean SUV measurements in VOI’s contoured for 65 lesions, grouped by those with volumes larger and smaller than 1 cm^3^. The CNN had difficulty to accurately quantify lesion SUVs, and this effect was amplified for small lesions in noisy data. Error bars denote the corresponding means of the intra-VOI standard deviations

Although the CNN quantification of large lesions was generally accurate, this was not found to be the case for small lesions, especially in noisy data where the network imposed a greater degree of suppression of high spatial frequencies. For the CNN-denoised images, overall lesion contrast recovery was 60% and 90% at the 1 and 20 million count levels, respectively; when including only those lesions smaller than 1 cm^3^, these numbers dropped to 41% and 85%. These effects were less pronounced in the original image set, and the VOI measurements for all count levels are displayed in Fig. 3—the data are grouped by volume to illustrate the effect of lesion size. As seen here, when lesion size decreased and image noise increased, contrast recovery suffered relative to the original images. This however, as reported in the “Lesion detection” evaluation section below, did not directly translate to poorer lesion detectability, which is more closely related to lesion signal-to-noise, rather than simply contrast [31].

**Fig. 4:**
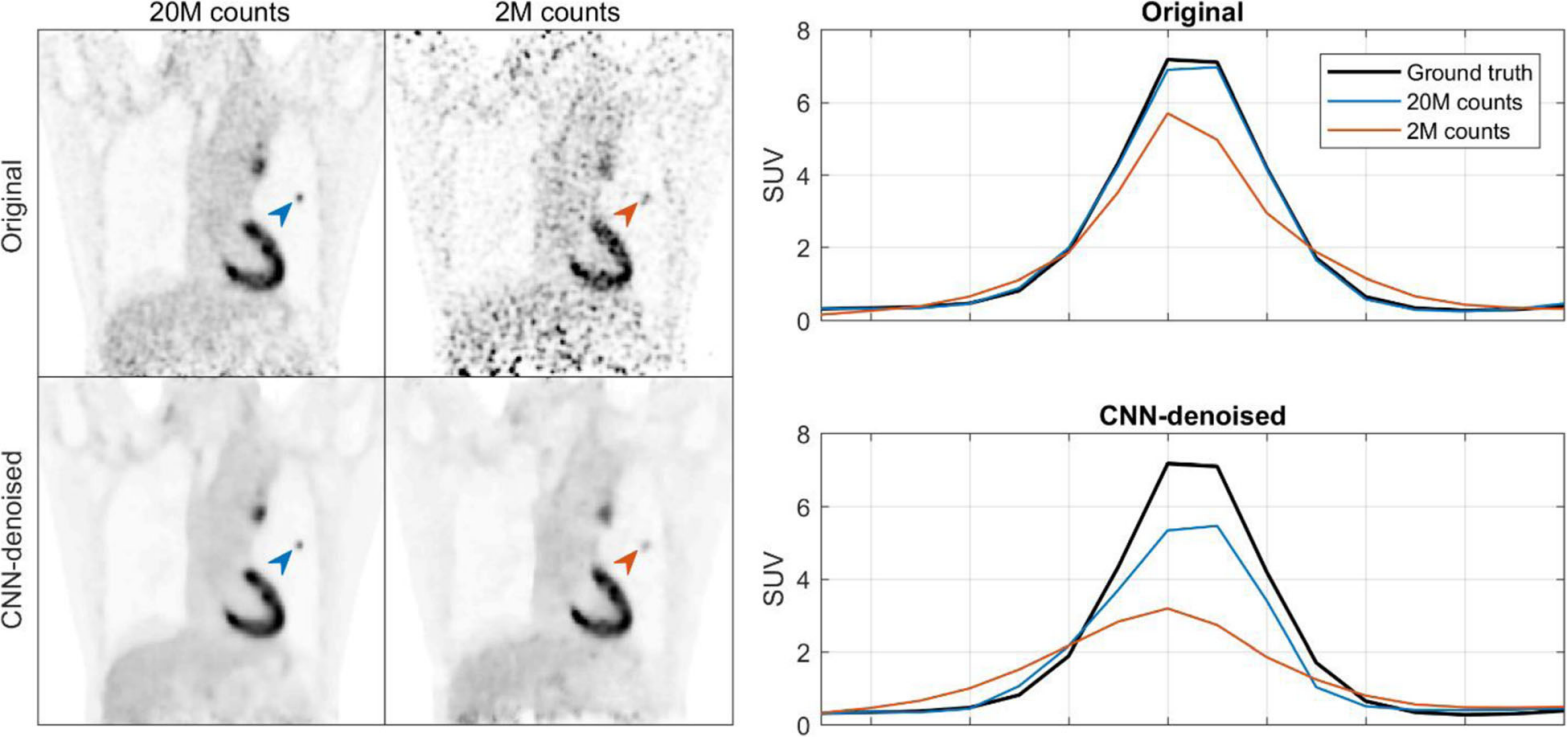
Example showing a small lesion (0.3 cm^3^) where its uptake was not fully recovered by the CNN

Figure 4 shows the activity profile for a small lesion in the lung parenchyma for 2 count levels, 2 and 20 million. In this case, neither CNN-denoised image was able to completely recover the contrast of the lesion.

### Qualitative evaluation

**Fig. 5:**
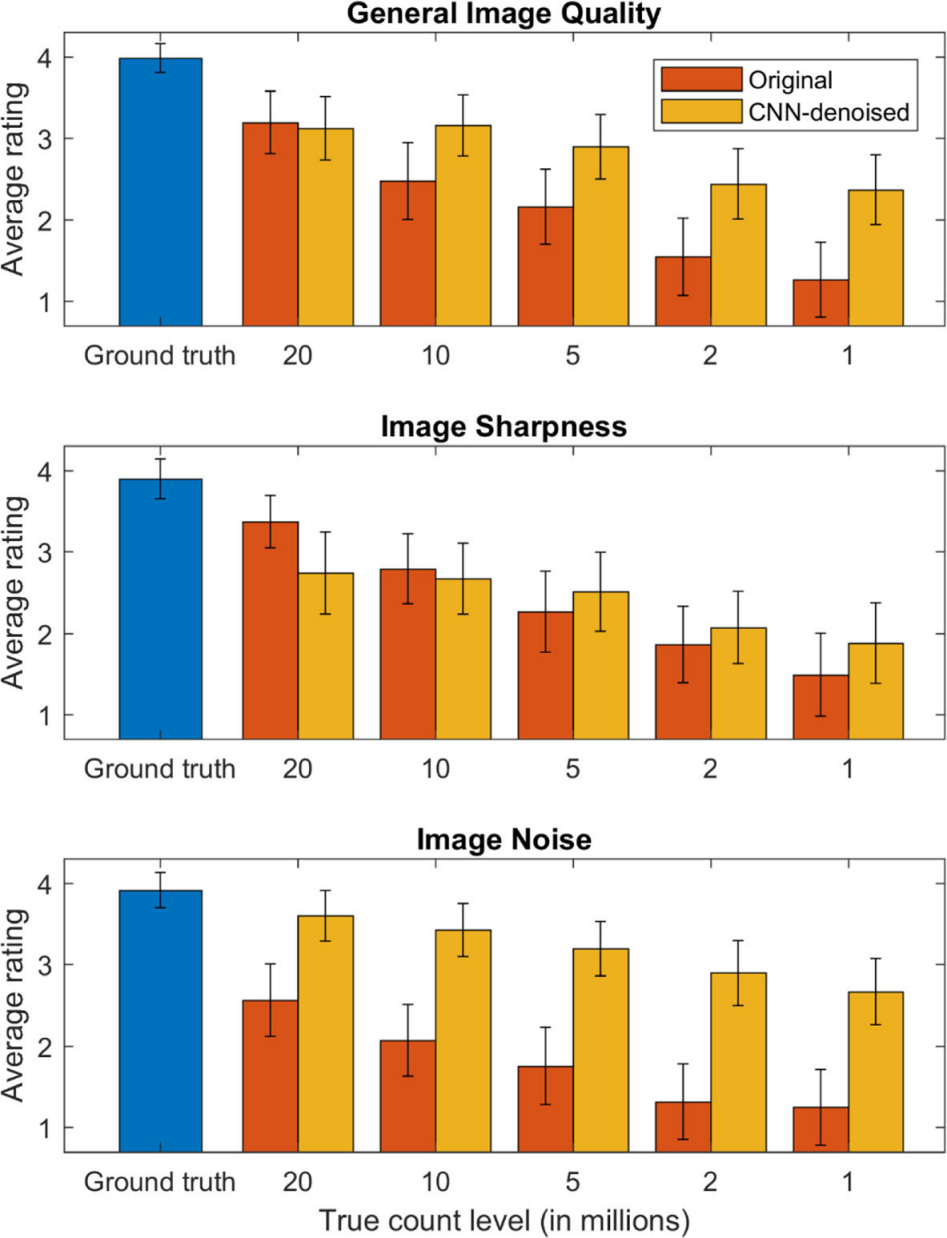
Image ratings determined by the 3 physicians in the reading task, pooled and averaged for each count level (higher is better). The standard deviation across all readers and images are indicated by the error bars

Regarding the clinical reading evaluation, three readers completed both phases of the task, and similar performance trends were observed among them. The results of all readers were pooled within each count level, averaged, and presented here, with inter-reader variability noted and any remarkable differences highlighted in the discussion. As seen in Fig. 5, with the exception of image sharpness, the CNN-denoised images were generally ranked equal to or better than the Gaussian-smoothed images for all count levels, with the largest effects observed in the lowest-count image sets. For example, at the 1 million count level, the average general image quality and image noise ratings were 1.26 and 1.25 for the original images and 2.37 and 2.67 for the CNN-denoised images, respectively.

### Noise reduction

**Fig. 6:**
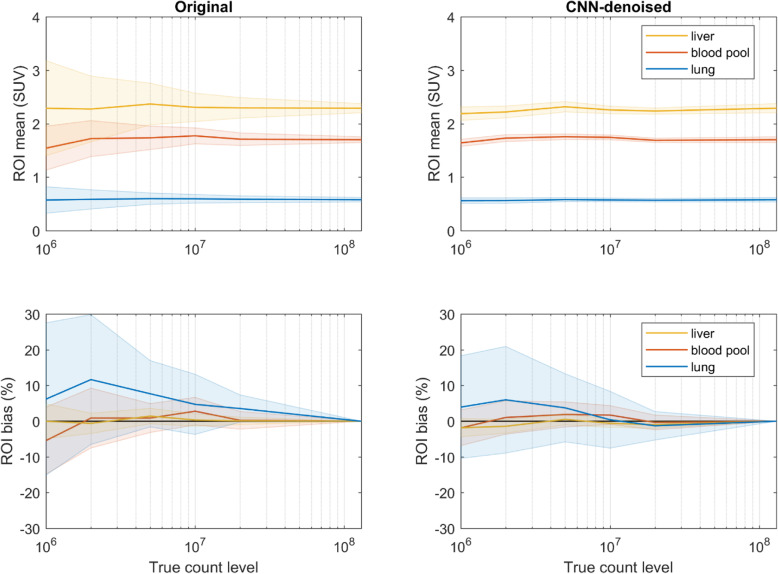
Voxel values for VOIs drawn over various tissue regions shown for all count levels. In the top row, the VOI means are denoted by the solid lines with the standard deviation of the VOI values represented by the shaded regions. The means are similar between the original and CNN-denoised images, but the measurement variance was significantly reduced in the latter set. In the bottom row, the mean VOI bias (relative to the full-count set) is denoted by the solid lines with the respective standard deviation shown by the shaded regions. VOI measurement bias in the CNN-denoised images was, in general, similar to or slightly lower than that in the original images. The lower observed variances in the mean and bias measurements suggest higher reproducibility in VOI measurements for CNN-denoising

The perceived improvements in image quality were largely dependent on the levels of image noise, i.e., the linear coefficient of determination for the rating differences in general image quality was 0.87 between those of image sharpness and 0.94 between those of image noise—this was also reflected in the VOI analyses. Figure 6 shows the VOI means and standard deviations, along with the corresponding VOI measurement bias relative to the full-count image sets. The VOI pixel variance was significantly reduced for all count levels in the CNN-denoised image set, especially in the noisiest data, e.g., at the 1 million count level, the mean standard deviation of the liver VOIs was 1.95 in the original images and 0.43 in the CNN-denoised images. The VOI measurement biases were somewhat similar, with the CNN-denoising images demonstrating slightly lower bias and corresponding variance in most cases.

### Lesion detection

**Fig. 7:**
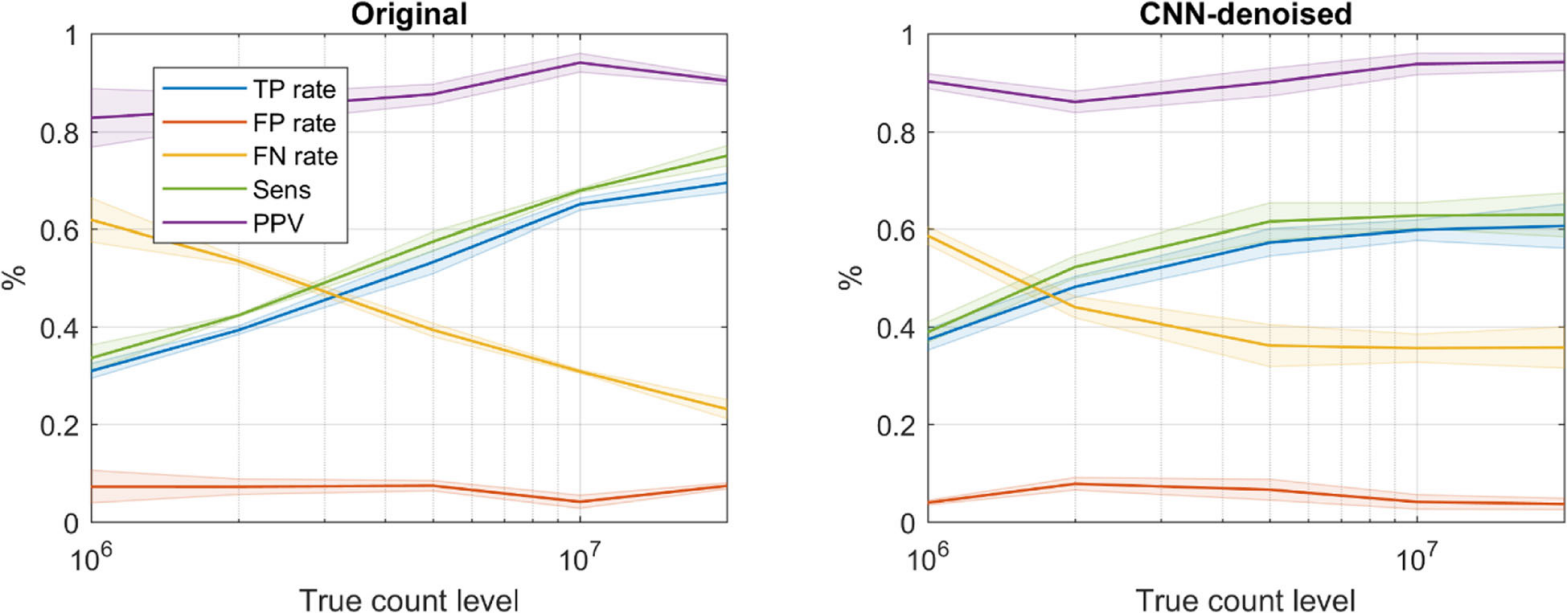
Lesion detection performance for all 3 readers. In the lowest count levels, CNN-denoising improved detection, but this offered limited benefit at higher count levels, and the original smoothed images actually yielded better performance. The observer mean is given by the solid lines, and the inter-observer standard deviation is shown by the shaded regions

Notwithstanding the reduced lesion contrast recovery in noisy data reported above, as seen in Fig. 7, the CNN-denoised images also yielded better lesion detectability in low count levels, i.e., data comprising 5 M or fewer counts. For example, at 1 million true counts, the average TPR was around 40% for the CNN-denoised images and 30% for the smoothed images. However as expected, as the true-count level increased, the relative performance in the CNN-denoised images waned, and the original images yielded better performance for the highest count levels. At 20 million true counts, average TPR and FNR were 60% and 35% for the CNN-denoised images and 70% and 20% for the original smoothed images. Sensitivity was strongly correlated with TPR. FPR and PPV improved somewhat with true count level but were less sensitive to post-processing method.

**Fig. 8:**
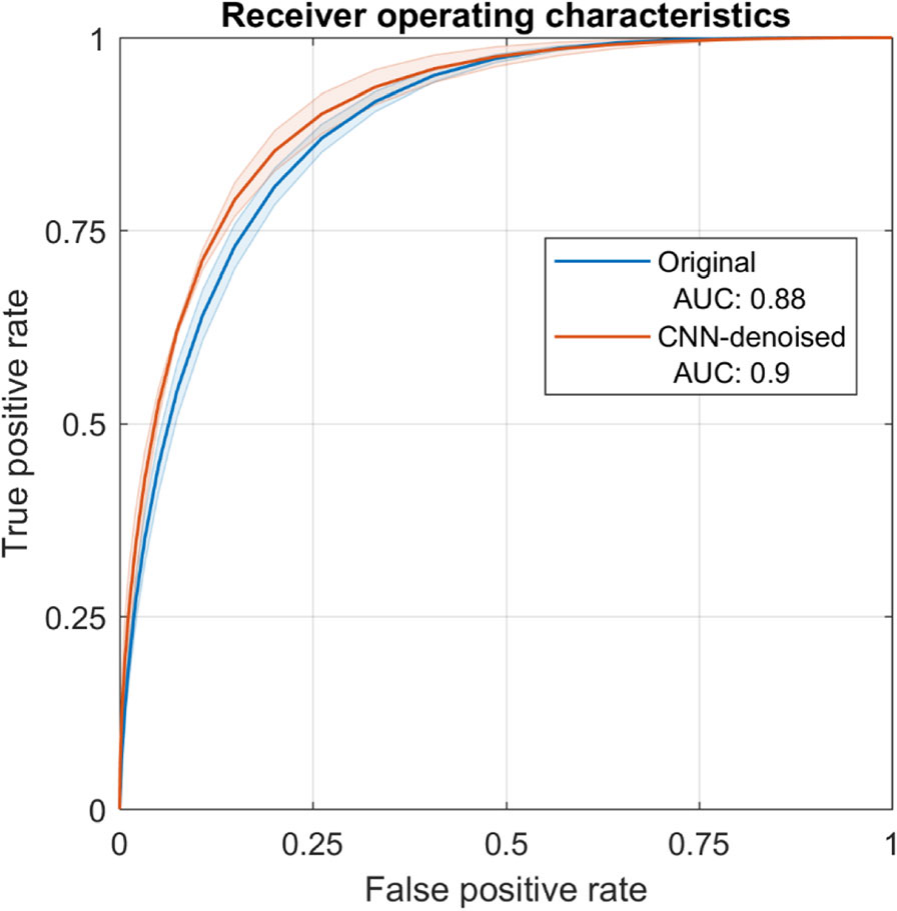
Receiver operating characteristic curves estimated from the confidence-weighted detections of the physician readers—again, the observer means are given by the solid lines, and the shaded regions denote the inter-observer standard deviation. Small overall differences were found between performance within the original and CNN-denoised images

Additionally, the ratings of every detection were used as a surrogate for observer confidence, by which receiver operating characteristic (ROC) analyses was performed [32]. Regarding the area under the ROC curve (AUC) in Fig. 8, only slight improvement was found overall for the CNN-denoised images (0.02)—the detection confidence ratings were generally higher within this set.

## Discussion

This work investigated the capacity for a 3D CNN to improve PET image quality and evaluated its impact within a clinical task-based framework—it found several advantages of CNN-denoised images over conventional Gaussian-smoothed images, especially at count levels below 5 M counts.

There are several novel aspects of this study. The majority of other related work has been performed in 2D since processing times are faster, fewer GPU memory issues are encountered, and the availability of pretrained 2D networks provides additional options in the choice of training objectives [23–26]. However, volumetric 3D PET data are the medical standard, and inclusion of the additional dimension of data was expected to improve training stability and robustness of the network performance [27]. Furthermore, to our knowledge, no previous work has evaluated the impact of CNN denoising within the context of a physician reading task—to this end, many groups have reported substantial improvements of the technique [24, 25], but few have noted any potential pitfalls. Finally, it was determined important from the beginning, to perform the experiment across a wide range of noise conditions to cover all realistic situations and to understand the limits of any potential benefits.

Consistent with previous studies [25, 26, 33], the findings presented here suggest that image quality, in terms of interpixel spatial variance and measurement reproducibility, were significantly improved. However, we found that these findings might not always translate directly into clinical improvements—the uptake in small foci was sometimes not accurately quantified, and lesion detection and localization realized less benefit. For example, in extremely high noise, the CNN-denoised images yielded better detection performance. At routine noise levels, however, performance suffered, and this might be attributed to the contrast recovery in small lesions. The network used in this study was trained with the L2-norm loss function, which is known to introduce slight blurring in the network output [16, 34, 35] and may be responsible to some degree for the limited recovery of lesion contrast. However, relative to other imaging modalities, PET already has intrinsically poor spatial resolution, and the comparison images were smoothed after reconstruction anyways, and so this argument might not account for the degree of inaccuracies observed. We surmise that the additional reason for the limited contrast recovery is that the network learned to suppress high frequencies in the input image data over a range of noise levels. For smaller lesions and increased background noise, it becomes difficult for the network to differentiate lesion signal from noise. We included in this study many noise levels to cover all realistic situations, but training with data specific to the target noise level could focus the objective and improve network performance. The introduction of a coregistered, anatomical correlate as an additional input to define morphological boundaries could also help to improve quantification accuracy.

Regarding the performance in the reading task, the overall trends among the physicians were similar. We did, however, notice that the most experienced of the three readers never ranked the CNN-denoised images with the highest rating, even at the highest count levels. This may be because the impressions of the images were not consistent with the previous clinical experience. This was also evident in the lesion detection task of the same observer, which showed lower performance in the high-count, CNN-denoised images relative to the other 2 physicians. This point highlights another important consideration concerning the clinical adoption of new technology, namely, there might be a transitionary period needed to become accustomed to the new appearance of otherwise familiar images. We also evaluated the relationship between detectability and lesion SNR; the latter was generally higher in all images denoised by the CNN. In the noisiest images, the lesion SNR was well correlated with improved detectability. However, in the higher-count images, it was observed that CNN-denoising also yielded higher lesion SNR measurements but lower detectability.

Notwithstanding potential pitfalls of using deep learning techniques to improve PET image quality mentioned here, we expect that this class of processing methods will still find its place in the medical setting. Specifically, it could prove useful for tasks involving noisy data, where absolute uptake quantification is unnecessary. One such future application might be PET for lung cancer screening, where a low-dose acquisition would be desired [36]—even if the uptake of small lesions was not accurately quantified, the overall improved noise characteristics could help to improve the identification of suspicious foci in images reconstructed from sparse data acquired by low-dose protocols. Other low-count situations which might benefit from this include pediatric imaging protocols where scan time and dose are constrained, dynamic imaging with fast temporal framing and physiological gated studies. More generally speaking, the clinical adoption of CNN-denoising will require careful consideration and planning and will depend on the imaging goals.

Some potential limitations of this study are noted. Nine patients were used for training—since end-to-end training of deep convolutional networks typically involves thousands of datasets, this number could seem largely insufficient. However, most patients had 2 different bed position acquisitions, and from each full dataset, around 270 unique images were realized. From each image, multiple smaller training patches were extracted at random. Hence, thousands of different training samples were produced at every training epoch. Even though the underlying activity distributions were limited by the patient population, this approach provided a clear objective for the CNN to learn the features of the image noise associated with the PET acquisition and reconstruction processes. Successful deep network training has also been reported in other work using a relatively small number of patient datasets [37]. Notwithstanding all of this, it was a major consideration in this study—although we carefully monitored the loss performance within the set of validation images in order to avoid overfitting, we expect accuracy and lesion detectability performance may be improved by including more patients for training.

The number of patients was also a consideration regarding bias in the lesion detection task, since an observer may have recognized a previous image at a different noise level. Although it may be impossible to eliminate this limitation entirely in a study like this, we sought to minimize it by randomizing the presentation order. This way, even if bias did exist, it would hypothetically affect both testing populations equally, and the comparison should still be valid. Also, the lesion detection analysis was based on a free response task, i.e., there was no limit to the number of lesion locations the observer was allowed to report in each image. So even if the observer recognized an image as a higher noise version of one they had already seen, it would be challenging to use any prior information to replicate every detection decision.

We did not intend to investigate the most advanced deep learning methods in this experiment. The goal of this study was instead focused on the clinical implications in a small, focused experiment with an established CNN architecture and relatively simple supervised training—in fact, PET data lend themselves very well to this approach. However, performance improvements could be expected by replacing the pooling layers with multiphase decomposition layers [38] in our network or by using additional loss objectives. Although a perceptual loss might not suit training a 3D network, a second discriminative loss network could provide higher quality outputs in the generator network. We chose for this experiment to compare the CNN-denoised images to the original images post-processed by Gaussian filter smoothing, since we involved physicians for the evaluations, and Gaussian smoothing is currently the clinical standard. It was beyond the current scope to compare to other smoothing techniques, even though more recent approaches, e.g., block-matching 3D [39] or spatially guided non local mean [40] algorithms, might produce better results. We also note that the conclusions drawn in this study are relevant to the current generation of PET systems. The mCT used in this study had 4 mm LSO crystal detector elements coupled to photomultiplier tubes—the coincidence timing resolution is around 550 ps. As detector technology, electronics and processing methods improve, higher quality data will be acquired, and better images will be reconstructed from the same underlying activity distributions. We evaluated here the quality of PET images only covering the lungs, and hence, the detection task focused primarily on pulmonary lesions. Such evaluations of noise and lesion detectability involving nodules surrounded by a cold air background are not equivalent to those for bone lesions in moderate background or small mesenteric nodes adjacent to surrounding loops of bowel, for example. The extensions of these analyses to other body regions and with more advanced architectures, loss metrics, and adversarial training techniques are opportunities for future work.

## Conclusion

In terms of image quality, the CNN-denoising offered several improvements over the original images—they exhibited better noise properties and improved measurement reproducibility of pixel values. In general, these images were also consistently ranked higher than the original low-count images in the clinical evaluation. They were also superior in terms of lesion detectability for data comprising 5 million or fewer true counts. However, CNN denoising offered limited improvements for the less noisy images and might not offer significant benefits in detection performance at the count levels routinely encountered in the clinic. To this end, there may be potential pitfalls of incorporating deep learning approaches into the medical setting which should be understood, regardless of apparent qualitative improvements. Considering this, the appropriate application of this technique in the clinic would depend on the task and the intended use of the output images.

## Data Availability

The patient data used in this study cannot be shared.
